# Neuromechanical activation of triceps surae muscle remains altered at 3.5 years following open surgical repair of acute Achilles tendon rupture

**DOI:** 10.1007/s00167-021-06512-z

**Published:** 2021-03-03

**Authors:** Markus Wenning, Marlene Mauch, Albrecht Heitner, Johannes Lienhard, Ramona Ritzmann, Jochen Paul

**Affiliations:** 1 Rennbahnklinik, Muttenz, Basel Switzerland; 2 Department of Orthopedic and Trauma Surgery, Faculty of Medicine, University Medical Center Freiburg, Albert-Ludwigs University of Freiburg, Freiburg, Germany; 3 Department of Sport and Sport Science, Biomechanics and Motor Control, Albert-Ludwigs University of Freiburg, Freiburg, Germany

**Keywords:** Achilles tendon rupture, EMG, Neuromuscular activation, Functional performance testing

## Abstract

**Purpose:**

To assess whether the neuromuscular activation pattern following Achilles tendon rupture and repair may contributes to the observable functional deficits in this severe and increasingly frequent injury.

**Methods:**

In this study, the neuromuscular activation using surface EMG of *n* = 52 patients was assessed during a battery of functional performance tasks to assess potential alterations of muscular activation and recruitment. We analyzed the injured leg vs. the contralateral healthy leg at a mean of 3.5 years following open surgical repair. The testing battery included isokinetic strength testing, bipedal and single-legged heel-rise testing as well as gait analysis.

**Results:**

During isokinetic testing, we observed a higher activation integral for all triceps surae muscles of the injured side during active dorsiflexion, e.g., eccentric loading on the injured leg, while concentric plantarflexion showed no significant difference. Dynamic heel-rise testing showed a higher activation in concentric and eccentric loading for all posterior muscles on the injured side (not significant); while static heel-rise for 10 sec. revealed a significantly higher activation. Further analysis of frequency of fast Fourier-transformed EMG revealed a significantly higher median frequency in the injured leg. Gait analysis revealed a higher pre-activation of the tibialis anterior before ground contact, while medial and lateral gastrocnemius muscles of the injured leg showed a significantly higher activation during push-off phase.

**Conclusions:**

The results of this study provide evidence on the neuromuscular changes 3.5 years following open surgical Achilles tendon repair. These complex neuromuscular changes are manifested to produce the maximum force output whilst protecting the previously injured tendon. The observed alterations may be related to an increased recruitment of type II muscle fibers which could make the muscles prone to fatigue.

**Level of evidence:**

III.

## Introduction

Achilles tendon rupture is a severe injury to any athlete and, regardless of the treatment, only about 70% achieve a return to preinjury athletic level [28]. Furthermore, of those that return, many show a reduced performance level in the first year after returning to sport or even longer [28, 29].

The reasons behind these performance deficits may primarily be found in the tendon healing itself; however, the neuromuscular impact on these deficits has rarely been investigated. Primarily an increased muscle activity and activation ratio on the injured side has been described during various tasks [17, 20, 26]. Further analysis has shown that the repaired Achilles tendon is compliant, but that greater strain results in less effective energy storage, e.g., during hopping tasks [20]. Additionally, studies reported less plantarflexion and increased dorsiflexion during gait which was linked to tendon stiffness and elongation [1, 9, 21]. An altered neuromuscular activation pattern reflected by the augmented tibialis anterior muscle and soleus muscle co-contraction index has been shown to be present [9]. Thus, it may be assumed that these alterations of neuromuscular activation contribute to the persisting mid- and long-term functional performance deficits [17]. While the structural changes observed in the tendon’s properties have been well investigated [22, 23], current literature underscores the importance of taking additional neuromuscular alterations into account when investigating persisting performance deficits after Achilles tendon rupture [14, 17, 25].

Thus, the aim of this investigation was to provide evidence for mid-term neuromechanical alterations following open Achilles tendon repair using a combination of performance tests in a large and representative cohort. Deducted from the literature [17, 20], it was hypothesized that muscular activation of plantar flexors would be higher in the injured leg compared to the healthy leg across all tasks, while neuromuscular activation pattern would be adapted to decrease excessive load on the ruptured tendon.

## Materials and methods

This is the second part of results from a large and multivariate cross-sectional comparative study. The study was performed in accordance with the Declaration of Helsinki, it was approved by the local ethics committee (EKNZ 2017-02206) and all participants declared informed consent prior to inclusion.

Mid-term neuromechanical activation deficits were assessed during functional performance testing of *n* = 52 patients which had previously undergone open surgical repair for acute Achilles tendon rupture. Inclusion criteria were acute Achilles tendon open repair in one of the two centers involved in the study, fewer than ten days after rupture, male gender and age < 60 years. Exclusion criteria were other injuries to the lower extremities, injury to the contralateral tendon, re-rupture, neurological impairments and diabetes mellitus. All tests were performed in a single session and supervised by two examiners experienced in biomechanical testing. The functional performance testing including strength testing, heel-rise test and gait analysis has been discussed separately [32].

### Patients

A total of *n* = 138 patients from two centers meeting the inclusion/exclusion criteria were assessed for eligibility. In summary, we were able to include *n* = 52 male patients at a mean follow-up of 3.45 ± 1.4 years, with a mean height of 1.81 ± 0.6 m and mean weight of 88.7 ± 11.7 kg. Mean age at surgery was 41 ± 9.5 years with the dominant leg (the leg one would jump with) injured in 52% of cases. The details of the recruitment process have been described before.

All patients had received physical therapy and followed a standardized rehabilitation protocol. All patients had finished their rehabilitation before enrollment in the study and had returned to any type sportive activities including running.

### Testing protocol

The details of the testing protocol have been described before [32]. The testing protocol included three different components:

(A) an isokinetic strength testing of plantarflexion and dorsiflexion (Humac Norm, CSMi, Stoughton, MA, USA) according to the literature [2]. The testing was performed with the patient placed in prone position in full knee extension. Three warm-up trials were followed by two sets of five repetitions at maximum effort. The protocol of concentric–concentric contractions at 30°/s angular speed in the full range of motion (ROM) was chosen due to its high test–retest reliability [2] and validity [18].
(B) Heel-rise testing using a novel approach in a marker-based 3D motion analysis laboratory (Vicon Motion System Ltd., Oxford, UK) and four different testing modalities: single-legged and bipedal testing, each during two different tasks: static testing with the patient staying at maximum heel-rise height for 10 s and dynamic testing with five repetitive heel-rises at moderate speed.
(C) Gait analysis using the same marker-based system for triggering EMG analysis via an integrated force plate (Kistler AG, Winterthur, Switzerland). Surface EMG Ag/AgCl electrodes (Blue Sensor Typ N, Ambu GmbH, Bad Nauheim, Deutschland) were placed on both shanks according to the SENIAM guidelines [12]. Transmission was realized using wireless EMG signaling (myon AG, Schwarzenberg, Switzerland) at a sampling rate of 2 kHz. EMG signal was collected bilaterally from the tibialis anterior muscle (TA), Gastrocnemius lateralis (GL) and medialis (GM) and soleus (SOL) muscle.

Details of the performance testing during EMG analysis were as follows:

*Isokinetic testing* Of the ten repetitions in total, the first one of each set was excluded to achieve a comparable preconditioning, the mean of the best four of the remaining eight repetitions was included in the analysis.

*Heel-rise* During heel-rise testing the time normalization was performed using the marker-based motion signal and the maximum and minimum value of the heel-marker defining the phases during dynamic heel-rise testing. For the analysis of muscle activity, we defined the first 2 s as the starting phase, a stable isometric phase of 6 s before entering into the final phase from seconds 8–10. We implemented a Fast Fourier analysis to measure muscle fatigue during the static condition.

*Gait analysis* During gait analysis, the pre-activation was defined as the integral activity 100 ms before ground contact. The Braking phase was defined from ground contact to when the anteroposterior force component at the force plate was crossing zero. Push-off phase was defined from the same point to toe-off. Mean value of six repetitions was calculated. Co-activation was defined as the normalized EMG of the plantarflexors (SOL, GM, GL) divided by the normalized EMG of the dorsiflexor (TA) [24].

### EMG signaling and processing

Bipolar Ag/AgCl surface electrodes (Ambu Blue Sensor P, Ballerup, Denmark, diameter 9 mm, center-to-center distance 34 mm) were placed over the tibialis anterior muscle (TA), gastrocnemius medialis muscle (GM), gastrocnemius lateralis muscle (GL) and soleus muscle (SOL) muscles of both legs. Procedures were executed according to SENIAM [12]. Post processing was performed using Nexus 2.7 (Oxford Metrics, Ltd., Oxford, UK), proEMG 2.0 (Prophysics, Kloten, Switzerland) and MathLabR2017b (The Mathworks Inc., Natick, MA, USA). Before statistical analysis, we applied a fourth-order butterworth bandpass filter (20–500 Hz) to a rectified EMG signal. EMG signal was integrated, time-normalized and normalized to MVC conditions according to the literature [5, 33] using the peak signal during maximum isokinetic contraction for amplitude normalization. To assess the firing frequency, a fast Fourier transformation (FFT) for spectral analysis was performed according to the literature with rectangular windowing and 2^*n*^ sampling of the median frequency [6, 30].

### Statistical analysis

The statistical analysis was performed using SPSS 24 (SPSS inc., Chicago, USA). Graphical display was realized using Veusz v. 3.0.1 (Veusz Group by Sanders et al., GNU-licensed, 2018).

An a priori sensitivity analysis assuming a power of 0.8 and an alpha error of 0.05 and a population of *n* = 52 resulted in a required effect size of 0.39. All variables were normally distributed (Shapiro–Wilk). The absolute values of the subgroups and the corresponding statistics are available in the online supplement. We performed a single-factor ANOVA with the factor side. For analyzing static heel-rise, we used a two-factor repeated-measures ANOVA with the three-level factor time and the two-level factor side with a Greenhouse–Geisser correction because Mauchly’s test of sphericity was significant for the factor time. Additionally, a post hoc analysis of pairwise comparison for the three-level factor time was performed. Generally, due to the explorative approach, the level of significance was kept at *p* < 0.05 for all tests, except post hoc analysis of rm-ANOVA where a Bonferroni–Holm correction of the alpha level was applied.

## Results

### Isokinetic testing

**Fig. 1:**
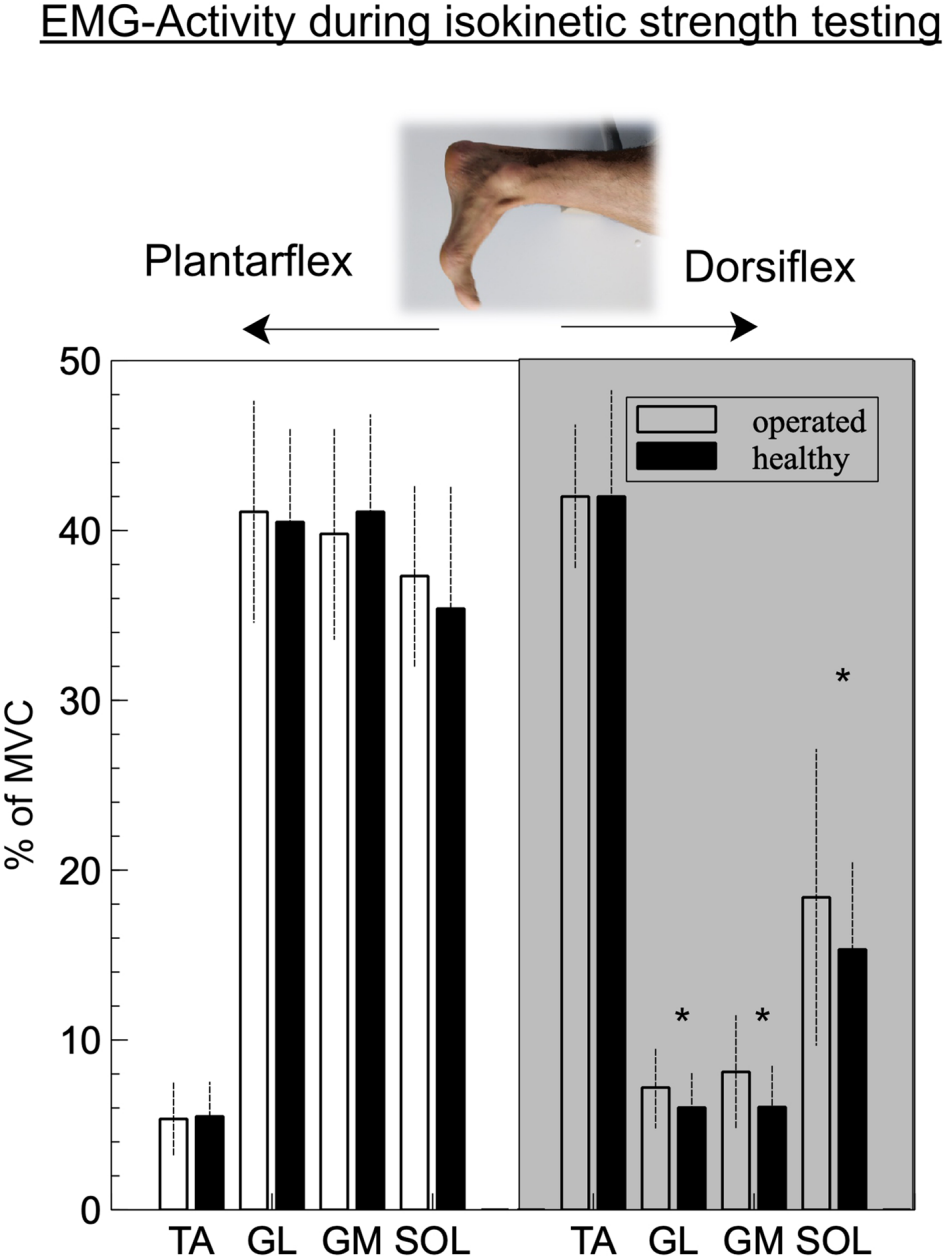
Muscle activation during isokinetic strength testing showing the increased muscular activation of the posterior muscle group during dorsiflexion. *p < 0.05

Isokinetic testing showed significant differences during eccentric activation when performing dorsiflexion with all muscles of the triceps surae complex showing a significantly higher activation rate on the operated side (Table 1, Fig. 1). The strongest effect was visible for gastrocnemius medialis muscle activation with an activation rate which was a relative 35% higher compared to the healthy leg (*F*(1,94) = 12,450, *p* < 0.001). The tibialis anterior muscle activation was comparable in both conditions. During dorsiflexion the activation of soleus muscle was nearly twice the amount, when compared to the two gastrocnemius muscles; however, this difference was not significant.

**Table 1:** EMG activation during single-legged isokinetic testing in concentric–concentric mode

Parameter	Muscle	Operated (%MVC)	Non-operated (%MVC)	ANOVA
Plantarflexion	TA	5.3 ± 2.1	5.5 ± 2.0	n.s.
	GL	41.1 ± 6.5	40.5 ± 5.5	n.s.
	GM	39.8 ± 6.2	41.1 ± 5.7	n.s.
	SOL	37.3 ± 5.4	35.4 ± 7.2	n.s.
Dorsiflexion	TA	41.7 ± 4.2	41.7 ± 6.2	n.s.
	**GL**	**7.2 ± 2.3**	**6.0 ± 2.1**	***p = 0.012***
	**GM**	**8.1 ± 3.3**	**6.0 ± 2.4**	***p < 0.001***
	**SOL**	**18.5 ± 8.7**	**15.3 ± 5.1**	***p = 0.03***

Bold values represent significant difference

*TA* tibialis anterior muscle; *GL* gastrocnemius lateralis muscle; *GM* M. gastrocnemius medialis muscle; *SOL* soleus muscle; *%MVC* in % of the peak value during maximum voluntary isokinetic contraction; *ANOVA* single-factor ANOVA, factor: side**,** mean ± standard deviation

### Heel-rise testing: dynamic

**Fig. 2:**
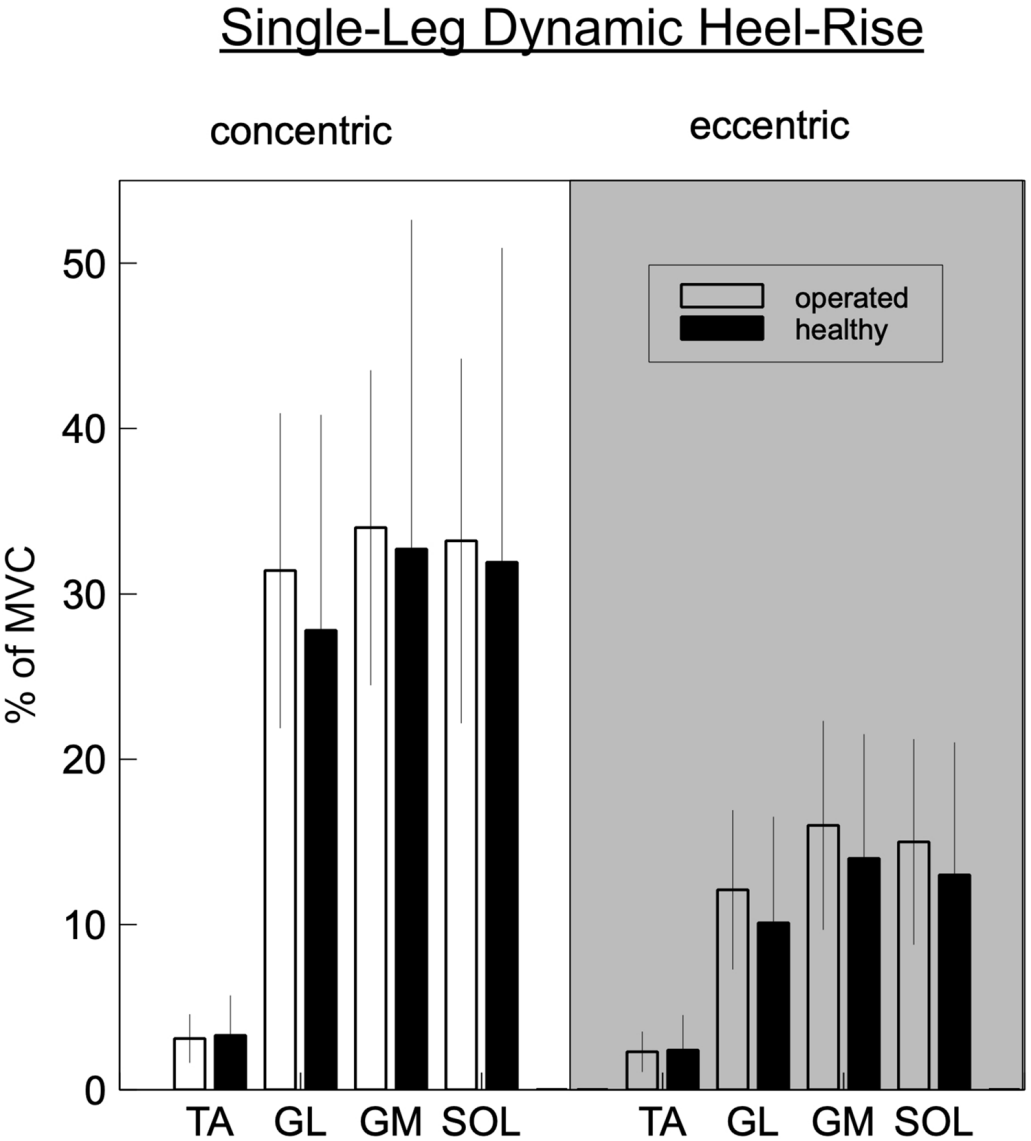
Muscle activation during single-legged dynamic heel-rise showing an increased integral muscular activity on the operated leg

The results of the dynamic single-leg heel-rise testing are displayed in Table 2 and Fig. 2; additionally in Table 2, the results of bipedal heel-rise testing are displayed. The activation rate of all three posterior muscles was higher when compared to the healthy leg; however, only lateral gastrocnemius muscle during eccentric contraction (“going down”) showed a significant difference (*p* < 0.05, *F*(1,84) = 5408). Activation of tibialis anterior muscle was comparable during concentric and eccentric contraction, while activation of triceps surae muscles was two–three times higher during concentric contraction, which resulted in a higher co-contraction index compared to concentric contraction.

**Table 2:** EMG activation during dynamic heel-rise

Parameter	Muscle	Operated (%MVC)	Non-operated (%MVC)	ANOVA
Single-leg dynamic	TA conc	3.1 ± 1.5	3.3 ± 2.5	n.s.
	TA ecc	2.3 ± 1.1	2.4 ± 2.1	n.s.
	GL conc	31.4 ± 9.5	27.8 ± 12.9	n.s.
	GL ecc	12.1 ± 4.8	10.1 ± 6.4	n.s.
	GM conc	33.8 ± 9.5	32.7 ± 19.9	n.s.
	GM ecc	15.7 ± 6.3	14.2 ± 7.5	n.s.
	SOL conc	33.1 ± 11.4	31.9 ± 18.5	n.s.
	SOL ecc	14.5 ± 6.2	13.4 ± 8.1	n.s.
Bipedal dynamic	TA conc	2.7 ± 1.4	2.8 ± 2.0	n.s.
	TA ecc	4.1 ± 3.2	4.2 ± 4.1	n.s.
	GL conc	25.1 ± 9.4	21.6 ± 11.8	n.s.
	**GL ecc**	**6.2 ± 2.9**	**4.7 ± 3.0**	***p = 0.02***
	GM conc	30.8 ± 14.5	28.3 ± 18.3	n.s.
	GM ecc	9.9 ± 4.5	8.7 ± 4.4	n.s.
	SOL conc	25.6 ± 10.4	21.7 ± 12.4	n.s.
	SOL ecc	7.4 ± 4.1	5.8 ± 3.9	n.s.

Bold values represent significant difference

*TA* tibialis anterior; *GL* gastrocnemius lateralis muscle; *GM* gastrocnemius medialis muscle; *SOL* soleus muscle; *conc.* concentric phase; *ecc.* eccentric phase; *%MVC* in % of the peak value during maximum voluntary isokinetic contraction; *ANOVA* single-factor ANOVA, factor: side, mean ± standard deviation;* n.s.* non-significant

### Heel-rise testing: static

**Fig. 3:**
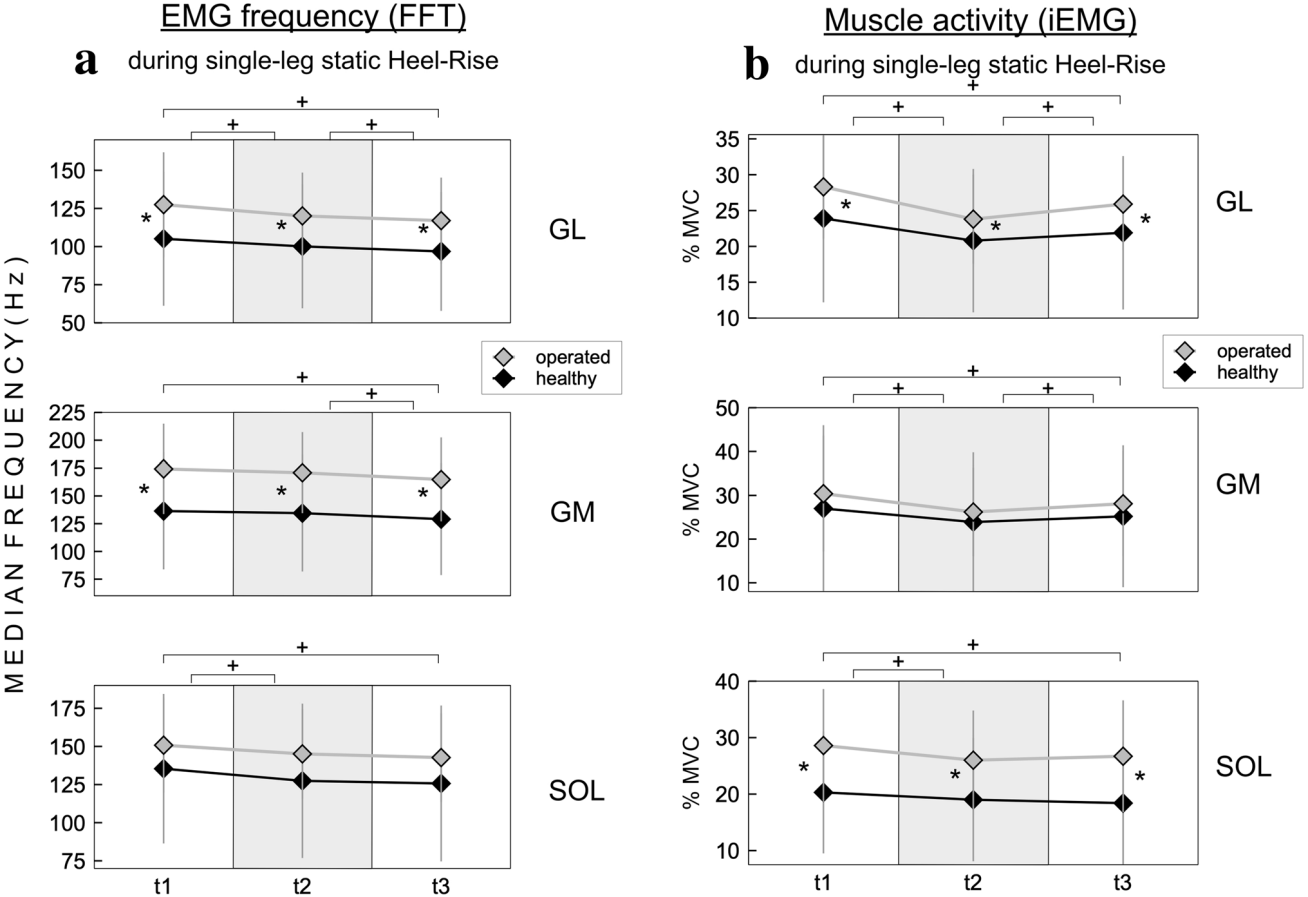
**a** Fast Fourier-transformed EMG during static heel-rise showing an overall increased frequency related to an increased recruitment in the activation of type II fibers on the injured side during static conditions. **b** iEMG during static heel-rise showing an overall increase in neuromuscular activation integral on the injured side. *p < 0.05 side-to-side difference, ^+^significant differences between time intervals *p* < 0.05. *t*1 = 0–2 s, *t*2 = 2–8 s, *t*3 = 8–10 s

Maximum amplitude and height during static heel-rise were significantly reduced in the injured leg. When performing single-leg static heel-rise testing, there were significant differences for time and side as shown in Fig. 3a + b and Table 3. The injured side showed a significantly higher integral EMG activity across all time intervals compared to the healthy side and the rm-ANOVA also revealed a significant effect of the factor time. The spectral analysis revealed a significantly higher median frequency in the injured leg compared to the healthy side during all time intervals for medial and lateral gastrocnemius muscle with strong effect sizes.

**Table 3:** EMG activation during single-legged static heel-rise testing

Muscle	Operated	Non-operated	rm-ANOVA	
			Factor time	OP vs. NOP
iEMG (%of MVIC)				
GL 0–2 s	28.3 ± 7.2	23.8 ± 11.7	*t*1 vs. *t*2 vs. *t*3: *p* < 0.001	*p* = 0.047
GL 2–8 s	23.8 ± 6.2	20.8 ± 10.0		
GL 8–10 s	25.9 ± 6.4	21.9 ± 10.7		
GM 0–2 s	30.4 ± 13.2	27.6 ± 18.9	*t*1 vs. *t*2 vs. *t*3: *p* < 0.001	n.s.
GM 2–8 s	26.2 ± 10.1	23.9 ± 15.9		
GM 8–10 s	28.0 ± 12.0	25.2 ± 16.2		
SOL 0–2 sec	28.6 ± 10.0	20.2 ± 10.8	*t*1 vs. *t*2 *p* < 0.001 *t*1 vs. *t*3 *p* < 0.001 *t*2 vs. *t*3 n.s.	*p* < 0.001
SOL 2–8 s	26.0 ± 8.8	19.0 ± 10.9		
SOL 8–10 s	26.8 ± 9.9	18.4 ± 10.9		
Median frequency in Hz (FFT-transformation)				
GL 0–2 s	127.5 ± 34.3	105.1 ± 43.9	*t*1 vs. *t*2 vs. *t*3: *p* < 0.001	*p* < 0.001
GL 2–8 s	120.1 ± 28.4	100.1 ± 40.6		
GL 8–10 s	117.0 ± 28.2	96.8 ± 38.9		
GM 0–2 s	174.3 ± 40.6	136.4 ± 55.6	*t*1 vs. *t*2 n.s. *t*1 vs. *t*3 *p* < 0.001 *t*2 vs. *t*3 *p* < 0.001	*p* < 0.001
GM 2–8 s	170.9 ± 36.4	134.4 ± 52.6		
GM 8–10 s	164.8 ± 37.7	129.1 ± 50.4		
SOL 0–2 s	150.8 ± 31.4	135.4 ± 49.0	*t*1 vs. *t*2 *p* < 0.001 *t*1 vs. *t*3 *p* < 0.001 *t*2 vs. *t*3 n.s.	n.s.
SOL 2–8 s	145.1 ± 29.4	127.4 ± 50.6		
SOL 8–10 s	142.7 ± 28.5	125.7 ± 51.1		

*OP* operated leg; *NOP* non-operated leg; *GL* gastrocnemius lateralis muscle; *GM* gastrocnemius medialis muscle; *SOL* soleus muscle; *%MVC* in % of the peak value during maximum voluntary isokinetic contraction; *FFT* fast Fourier transformation, 2^*n*^ of the median frequency; *rm-ANOVA* repeated-measures ANOVA**,** mean ± standard deviation; *n.s.* non-significant

### Gait analysis: EMG

**Fig. 4:**
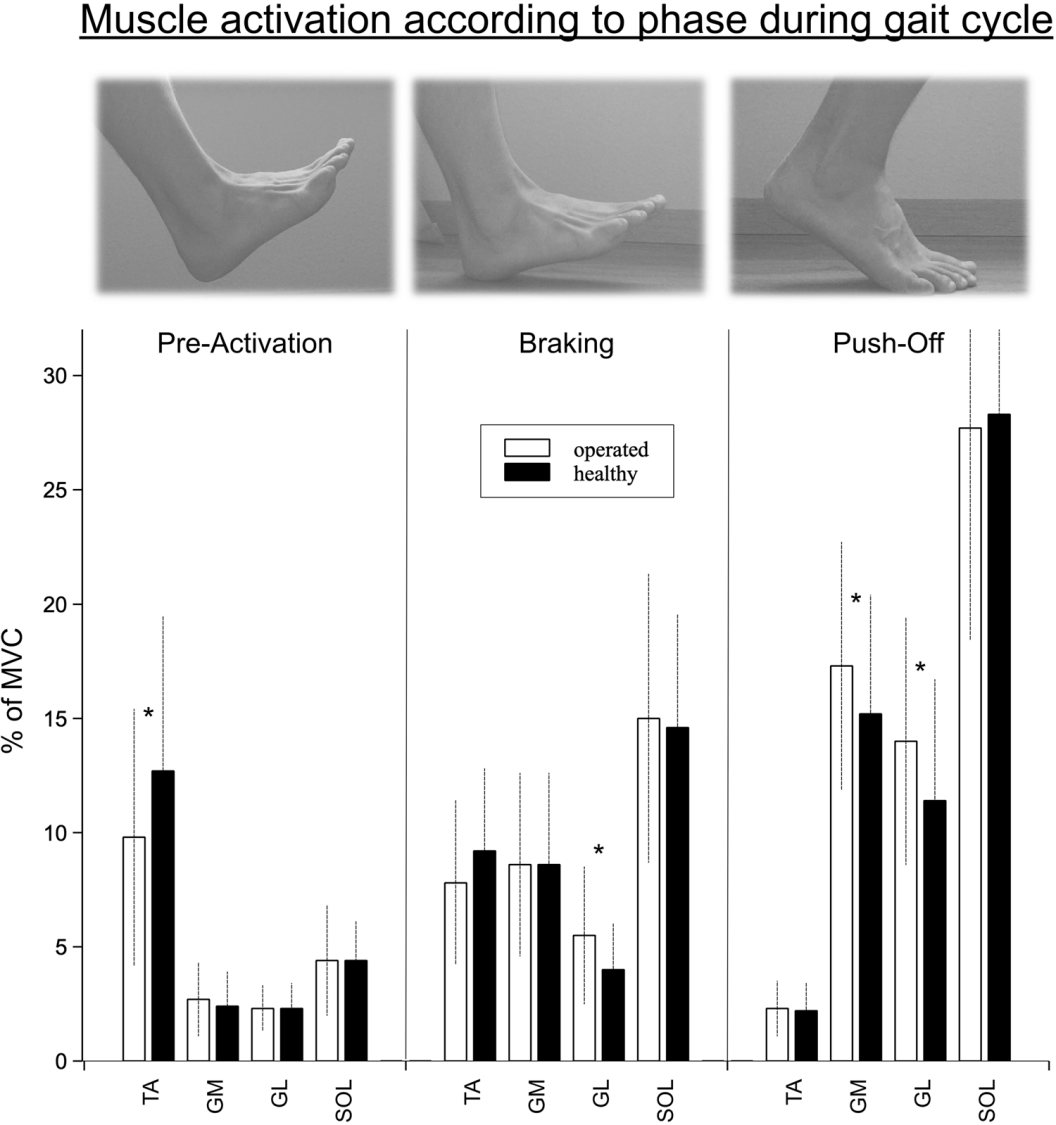
EMG activation pattern during gait analysis across the three phases. *p < 0.05

Gait analysis showed a significantly lower pre-activation 100 ms before ground contact for tibialis anterior muscle of the injured leg as displayed in Fig. 4. The co-contraction index of the injured ankle’s dorsi- vs. plantarflexors was, therefore, 1.05 compared to the 1.35 of the healthy contralateral leg. During braking and push-off phase, the lateral gastrocnemius muscle showed a significantly higher muscle activation on the injured side and, during push-off phase, this was also the case for the medial gastrocnemius muscle (Table 4).

**Table 4:** EMG activation during gait cycle

Muscle	Phase	Operated (%MVC)	Non-operated (%MVC)	ANOVA
TA	**Pre-activation**	**9.8 ± 5.6**	**12.7 ± 6.8**	***p = 0.026***
	Braking	7.8 ± 3.6	9.2 ± 3.6	n.s.
	Push-off	2.3 ± 1.2	2.2 ± 1.2	n.s.
GM	Pre-activation	2.7 ± 1.6	2.4 ± 1.5	n.s.
	Braking	9.8 ± 4.0	9.2 ± 4.0	n.s.
	**Push-off**	**17.3 ± 5.4**	**15.2 ± 5.2**	***p = 0.047***
GL	Pre-activation	2.3 ± 1.0	2.3 ± 1.1	n.s.
	**Braking**	**5.5 ± 3.0**	**4.0 ± 2.0**	***p = 0.005***
	**Push-off**	**14.0 ± 5.4**	**11.4 ± 5.4**	***p = 0.015***
SOL	Pre-activation	4.4 ± 2.4	4.4 ± 1.7	n.s.
	Braking	15.0 ± 6.3	14.6 ± 5.5	n.s.
	Push-off	27.7 ± 9.3	29.3 ± 9.7	n.s.

Bold values represent significant difference

*TA* tibialis anterior muscle; *GL* gastrocnemius lateralis muscle; *GM* M. gastrocnemius medialis muscle; *SOL* soleus muscle; *%MVC* in % of the peak value during maximum voluntary isokinetic contraction; *ANOVA* single-factor ANOVA, factor: side, mean ± standard deviation

## Discussion

The most important finding of this study was that during eccentric loading and isometric contraction, there is a higher activation of the triceps surae muscle complex on the injured side compared to the healthy contralateral side. Furthermore, the frequency analysis showed a higher recruitment on the injured side, which could be related to the increased activation of fast-twitch fibers. Finally, the activation pattern during gait revealed a significantly lower pre-activation of the tibialis anterior muscle before ground contact; while during the braking phase, there was a significantly higher activation of the lateral gastrocnemius muscle. During push-off phase, the activation of medial and lateral gastrocnemius muscles was also significantly higher, while soleus muscle activation was not altered.

### Isokinetic testing

While the recruitment pattern during active concentric plantarflexion was not altered between the legs, there was a significant difference during active dorsiflexion for all three posterior muscles (Fig. 1). This increased muscular activation, however, does not lead to a reduced maximum dorsiflexion torque, but may in part be the reason why maximum peak torque angle during dorsiflexion is altered [32]. Moreover, the increased activation of all triceps surae muscles suggests that “active facilitation” during dorsiflexion is not observable following Achilles tendon rupture [13, 20].

The causal interpretation of this observation requires further analysis since, among others, we suggest that the following factors may contribute: (1) maximum concentric voluntary activation may exceed the side-to-side differences and, therefore, mask potential differences related to muscular activation that otherwise would support the continuously increased general muscular activation [26, 33]; (2) the increased activation during dorsiflexion may be caused by an altered spinal reflex path due to impaired afference from the repaired tendon itself [1]; or (3) there is persisting co-contraction as a preventive measure to limit excessive e.g. ballistic contraction and subsequent tendon elongation. As discussed by other authors before, this co-contraction may prevent the tendon from explosive, eccentric loading, even though this activation pattern increases net tendon strain [4, 7, 27, 33].

McHugh et al. suggested that the increase in activation may be attributed to the decrease in muscle fiber length and in their study, this increase in activation was pronounced when there was a significant weakness during plantarflexion [17].

### Dynamic heel-rise testing

Furthermore, during eccentric contraction in dynamic heel-rise testing (e.g., “going downward”), there is an increased muscular activation as it has been described before even in healthy subjects comparable to our results [11]. These findings are in line with McHugh et al. where an increased muscular activation was necessary to perform the same motor task [17, 34]. Using a different setup, Zellers et al. found a comparable, yet statistically significant, increase in plantarflexor activation 100 ms before landing from a jump, which was attributed to controlling dorsiflexion and stiffing the joint prior to eccentric load [34]. The same pattern was observed during bipedal testing.

Interestingly, even though the weight in this task is partially shifted away from the injured side and consequently less force is required, the muscular activation level is still increased.

### Static heel-rise testing

As in all testing protocols, we found a higher neuromuscular activation in all time intervals on the affected side, which is in line with preliminary findings in literature [17]. The spectral analysis of EMG activation revealed a significantly higher median firing rate for GM and GL on the injured side, which has been linked to the activation of fast-twitch fiber group [6, 33]. This observation may either be attributed to a change in structural fiber type from slow-twitch to fast-twitch, which has been described for other injuries [10] or it may result from a different recruiting pattern, where an increased recruiting of type IIa/b fibers is necessary to produce sufficient isometric strength [3, 31]. The etiology of this should be the focus of future research. Regardless of the cause, this may serve as evidence that the contractile potential of the triceps surae muscles is reduced and the muscle could be more prone to fatigue.

### Gait analysis

The finding that the activation level of the plantarflexor muscles during push-off phase is significantly higher may indicate that the energy storage in the injured tendon during the eccentric phase is less effective so that compensatory muscle activation is necessary to produce comparable propulsory force [1, 16, 25]. It can be deducted from the literature that the deficit in tendon recoil work on the injured side must be compensated for by concentric muscular work and, thus, results in a higher activation level compared to the healthy tendon’s side [13, 15, 19].

It is of additional interest that the pre-activation of the tibialis anterior muscle 100 ms before ground contact is significantly lower on the injured side, while the posterior muscles exhibit the same pre-activation level in both legs. This observation may be attributed to the overall reduced tension of the muscle–tendon unit of the triceps surae muscle and potentially an elongation of the tendon, which consequently requires less activation of the TA to achieve the same joint position at ground contact. However, a recent review found that tendon elongation correlates with biomechanical parameters, but not patient-reported outcome, yielding that this requires further and detailed analysis [8].

Limitations of the study include the retrospective recruitment of patients, which always poses a risk of a recruiting bias. However, we have analyzed the non-recruited patients according to their biometrical data and we have not found any indication for a recruiting bias. Further limitations include the novel testing setup and the lack of causal investigation regarding tendon lengthening and stiffness. Thus, we are unable to report the tendon length and stiffness; however, earlier studies have not been able to underscore a correlation between tendon length and neuromuscular activation [25].

## Conclusions

The results of this study provide evidence that there are persisting neuromuscular adaptations 3.5 years after open Achilles tendon repair. These neuromuscular changes are manifested consistently for simple monoarticular up to complex whole-body multiarticular movements. The increased neuromuscular activity, thereby, precludes a primary phenomenon of neural inhibition as part of the performance deficit. The slightly increased muscle co-contraction indicates a neuromuscular stiffing of the joint to reduce the degree of freedom in favor of safety.

## Abbreviations

TA: Tibialis anterior muscle
GM: Gastrocnemius medialis muscle
GL: Gastrocnemius lateralis muscle
SOL: Soleus muscle
EMG: Electromyogram/-graphy
